# The Role of The Predominant Polarity on Neurocognitive and Social Cognitive Dysfunctions in Patients with Bipolar Disorder

**DOI:** 10.1192/j.eurpsy.2023.1078

**Published:** 2023-07-19

**Authors:** E. Atay, C. Ermis, E. Ozmen

**Affiliations:** 1 Psychiatry, Manisa Celal Bayar University Hospital, Manisa; 2 Child and Adolescent Psychiatry, Dokuz Eylul University Hospital, Izmir, Türkiye

## Abstract

**Introduction:**

Bipolar disorder (BD) is characterized by significant inter-individual variation in terms of course and outcome. The most important factors associated with the outcome are subsyndromal depression and cognitive disability. Predominant polarity (PP), which is a proposed course specifier for BD, can be associated with various clinical differences such as psychotic feature, suicidality, hospitalization, while it is thought to be associated with the severity of cognitive impairment.

**Objectives:**

To elucidate the role of the predominant polarity on cognitive dysfunction in patients with BD.

**Methods:**

Patients with BD in remission (n=84) and healthy control volunteers (HC, n=27) participated in the study. Patients were divided into 3 subgroups according to their PP characteristics: manic (MPP, n=31), depressive (DPP, n=25), and undetermined (UPP, n=28). Structured Clinical Interview for DSM-5 Disorders-Clinician Version (SCID-5/CV), WAIS-R-Vocabulary Subtest, Hamilton Depression Rating Scale, Hamilton Anxiety Rating Scale, Young Mania Rating Scale, Rey’s Auditory Verbal Learning Test (RAVLT), Trail Making Test (TMT), Stroop Test (ST), WMS-R Visual Reproduction Subtest, Controlled Oral Word Association Test (COWAT), Auditory Consonant Trigrams Test (ACT), Reading the Mind in the Eyes Test (RMET), Hinting Test (HT), Wisconsin Card Sorting Test (WCST), and Conners Continuous Performance Test (CCPT) were administered. Scores that do not show normal distribution were transformed using the two-step normalization method. Principal component analysis (PCA) with direct oblimin rotation was applied as a dimension reduction technique to identify different neurocognitive domains. Single-factorial PCA was also applied to calculate global cognition scores.

**Results:**

In MPP group compared to HC, worse performance was observed in ACT (d=1.24), COWAT (d=1.16), RMET (d=1.03), HT (d=1.78), WCST correct answers (d=0.99), CCPT correct target section (d=0.99) and a prolongation in TMT-A (d=1.00). Compared to DPP, MPP had a weak performance in COWAT (d=0.89), RMET (d=0.86) and HT (d=1.00). MPP (d=1.18) and UPP (d=1.03) groups showed deterioration in processing speed compared to HC. MPP group showed impairment in working memory (d=1.17) and attention (d=0.70) compared to HC. In problem-solving and reasoning, deterioration was found in MPP compared to HC (d=1.16) and UPP (d=0.67), also in DPP compared to HC (d=0.74).

**Conclusions:**

The MPP group yielded more severe cognitive impairment in verbal fluency and social cognition tests compared to DPP. Predominant polarity may also be related to cognitive impairment patterns seen in BD.

**Disclosure of Interest:**

None Declared

